# Proceedings of the Georgia State Medical Association

**Published:** 1872-04

**Authors:** Wm. Abram Love


### PROCEEDINGS OF THE GEORGIA STATE MEDICAL ASSOCIATION.

REPORTED BY WM. ABRAM LOVE, M.D.,

COLUMBUS, GEORGIA, April 10, 1872.

The Georgia State Medical Association convened in Columbus to-day, in a hall adjoining the Rankin House, and was called to order at half-past twelve o'clock, by the President, Dr. George M. McDowell.

The deliberations of the body were opened with prayer by Rev. Dr. Hunter.

Dr. V. H. Taliaferro, in behalf of the medical profession of the city of Columbus, bade the Association a cordial welcome, in a chaste, eloquent, and appropriate address. He said the members represented every section of the State. The object of the assembling was to promote science by collation of facts and interchange of views and discussions. The profession is so allied with benevolence and charity that all feel a common interest and sympathy with the deliberations and the practitioners. He hoped the session would be harmonious, and result in good. He referred to the results of the science in warding off coast epidemics, and anticipated the day when our cities, under a proper system of quarantine, might be as effectually shielded as all are now comparatively protected from small-pox (that once terrible scourge) by vaccination. He alluded to the wonderful advances made in medicine. In the last thirty years, chloroform and ether have been discovered; chloral, which supplies an important need, has been known only a few years; and electro-therapeutics, now in its infancy, promises to produce the happiest results. He also mentioned other brilliant discoveries. Questions of vast importance are before you. Great masters in the healing art have arisen, and will still be born. Cures for tuberculosis, cancer and scrofula may yet be discovered, and give great fame to American scientists. Reform in medical education is required. By many, from failures hitherto, it is thought the American Medical Association is incompetent to the task it has undertaken. In many sections, the charlatan competes successfully with the man of learning for patronage. Ignorance knows nothing of wisdom, and fosters ignorance. Common education of the masses may solve the question, and give scientic attainments their proper meed and status. He hoped the stay of the Association in the city would be pleasant and agreeable as a body and as individuals.

His Honor, Mayor McIlhenny was then introduced, who welcomed the body to the hospitalities of the city in a feeling and timely address. He said it gave him pleasure to welcome to the city such an association of learning, talent, and usefulness. The citizens of Columbus are glad to have them here. The city has many things which are of the highest interest, and many things that would please all, for they evinced the enterprise and skill of Georgians. The members would be entertained by visiting the cotton and other manufactories, the public library and schools, where they would be gladly welcomed. Columbus desired to have pleasant impressions carried to the homes of members. He extended to the Association a cordial welcome, and hoped the members would again honor the city by their presence.

In behalf of the Association, Dr. A. W. Griggs, of West-Point, in brief and happy style, returned thanks to the Medical Society and the city for the very cordial welcome extended, and the manner in which it had been done. From the known hospitality of the city, the Association expected kind treatment. The object of assembling was to advance science, and especially to elevate the science of medicine, and to awaken the sympathy and kindness of all with the medical fraternity. Though much was expected from Columbus, yet the members had received one delightful welcome which had not been anticipated, and that was the presence and bright smiles of the fair sex. He returned warmest thanks to them. The younger members of the profession—and older, too—would, on leaving Columbus, but lovingly think of "The girl I left behind me."

At the conclusion of Dr. Griggs' address, on motion of Dr. E. J. Kirkscey, of Columbus, the Association adjourned until three o'clock P.M., to allow members time to register their names with the Secretary.

AFTERNOON SESSION.

The Association was called to order at three o'clock P.M., by Dr. George M. McDowell, President.

The registration of members being in order, the following names were recorded:

*Officers.*—George M. McDowell, President, Barnesville; E. J. Kirkscey, Columbus, First Vice-President; W. A. Greene, Americus, Second Vice-President; S. H. Stout, Atlanta, Permanent Secretary; W. O'Daniel, Twiggs county, Treasurer; A. W. Griggs, West-Point, Orator.

*Members.*—R. B. Anderson, Roswell; John E. Bacon, T. F. Brewster, F. L. Brooks, Columbus; Robert Battey, Rome; W. R. Burgess, Macon; A. B. Calhoun, Newnan; T. P. Chaffin, J. L. Cheney, W. N. Coe, E. F. Colzey, Columbus; A. L. Connally, W. H. Cummings, Atlanta; E. L. Crump, Herndon; C. A. P. DeCortez, W. Duncan, Savannah; Edwin F. DeGraffenried, Columbus; W. H. Evans; John Fish, Chatham; William Flewellen, James W. Ford, Columbus; W. T. Goldsmith, W. H. Goodwin, Atlanta; W. A. Greene, Americus; T. G. Greene, Roswell; George E. Grimes, T. W. Grimes, Columbus; A. W. Griggs, West-Point; Charles H. Hall, D. W. Hammond, W. F. Holt, Macon; G. B. Heard, Columbus; Juriah Harris, Savannah; M. W. Harris, Perry; S. B. Hawkins, Americus; George C. Henry, West-Point; J. W. Herty, Milledgeville; Geo. N. Holmes, Macon; G. W. Holmes, W. D. Hoyt, Rome; E. H. W. Hunter, Louisville; C. T. Jelks, Marietta; J. T. Johnson, Atlanta; H. M. Jeter, E. J. Kirkscey, S. B. Law, Columbus; J. P. Logan, Wm. Abram Love, Atlanta; J. J. Mason, C. J. Moffett, Columbus; K. P. Moore, Knoxville; L. Morgan, Z. H. Morgan, Cedar Grove; George M. McDowell, Barnesville; Q. W. Norton, Savannah; Charles B. Nottingham, Macon; W. O'Daniel, Bullard Station; L. H. Orme, Atlanta; H. Perdue, The Rock; W. H. Philpot, Talbotton; G. W. Pitts, Columbus; W. F. Pendleton, B. L. Purse, Savannah; G. R. Powell, Milledgeville; Edwin S. Ray, Atlanta; R. B. Ridley, LaGrange; B. F. Rudisill, Forsyth; A. J. Shaffer, Lawrenceville; A. H. Shi, Colaparchee; C. D. Smith, Newnan; C. L. Smith; F. A.

Stanford, Columbus; S. H. Stout, Atlanta, Theodore Starbuck, Savannah; G. E. Sussdorff, Macon; J. G. Thomas, Savannah; V. H. Taliaferro, T. S. Tuggle, Carlisle Terry, Columbus; J. T. Todd, West-Point; J. A. Urquhart, T. J. Word, Columbus; J. Walker, Calhoun; John Stainback Wilson, W. F. Westmoreland, Atlanta; P. H. Wright, Macon, and others.*

The President delivered his address, the points of which we will endeavor to give. He said the Associations were pleasant reunions—mile-stones along the pathway of life to show how far we had gone. The course of this and similar Associations has been upward and onward, and results have been obtained far greater than could ever have been achieved by individual efforts. The effect has been a higher standard of medical culture, and should be made so. The profession requires a lifetime study, and gives little opportunity for preparatory education after practice has begun. A regularity in medical colleges and sincere observance of the Code of Ethics will greatly advance the profession. Such Associations have wonderfully improved medical and scientific journals of this country. He thought the plan of offering premiums for essays, which had fallen into disuse in this State, effected good. What is known as State medicine, quarantine, etc., should receive especial attention. The voice of the physicians of the State will have the weight of authority, and devise the best system. The mortality in some enlightened countries is as great as one hundred years ago, notwithstanding the advancement of science. The collection of vital statistics, study of climatology, and the removal of certain teachings which tend to demoralize society, should receive our careful attention. He advised a State Board of Health and compulsory vaccination, and approved the adoption, by the last session, of the Inebriate Asylum. Infidelity and atheism are making rapid strides, especially in Germany, and should be combatted. The test of all truth is not its seeming utility. The medical profession is not one for slothful and idle men, but for

*There were in attendance, as *members*, one hundred and thirty-three; but it was impossible for us to get a complete list of the names. There were also many visitors from Alabama and elsewhere.

faithful students, who keep up with scientific literature and discoveries.

On motion of Dr. S. H. Stout, the municipal authorities of the city, the clergy, and the representatives of the press, were invited to seats.

On motion of Dr. W. A. Love, regular members of the profession of this or sister States, visiting the city during the session, were invited to seats in the Association.

On motion of Dr. Stout, Dr. J. T. Johnson was requested to act as Assistant Secretary.

Drs. F. F. Brooks, T. P. Chaffin, E. F. Colzey, J. M. Foard, George J. Grimes, George Heard, S. B. Law, J. J. Mason, C. J. Moffit, H. Perdue, J. W. Pitts, B. F. Rudisill, C. D. Smith, Carlisle Terry, and T. J. Word, were elected permanent members of the Association.

The Committee on the Constitution was called, when Dr. Griggs requested further time (until to-morrow) to report. The committee was allowed further time.

The Committee on Vaccination, of which Dr. Logan was Chairman, was discharged.

The Committee on Publication reported, which report was received.

It was moved by Dr. Hunter, that a committee be appointed to memorialize the Legislature, and ask that the physicians' lien extend to all property not embraced in the homestead.

The report of the Committee on Inebriate Asylum was received and adopted.

Dr. Stout read a communication from Th. Schumann, pharmaceutist, of Atlanta, on the duties and relations of physicians and pharmaceutists to each other.

Dr. J. Harris made some remarks on the subject of the communication—on the repeating of prescriptions without the order of the physician—claiming that such restriction was rather a safeguard to all parties.

On motion of Dr. Stout, the communication was referred to the Committee on Publication.

Dr. W. A. Greene moved a suspension of the rules, for the election of permanent members, which motion was adopted.

Drs. E. M. Crump, C. A. P. DeCortez, W. I. Greene, —— Herndon, J. W. Norton, W. F. Pendleton, and Thomas Starbuck, were elected.

Dr. Robert Battey, of Rome, stated that there had been a disturbance long existing in the Association, which had served to engender strife and bitter feelings in the breasts of many members of the profession throughout the State; that it had tended to mar the peace and destroy the usefulness of the body; but that he was happy to state, that through the labors of many of the members and friends to the parties, these differences had been amicably adjusted outside of the Association, and forever buried, and he doubted not that all would join him in rejoicing at the consummation of so desirable an end. He furthermore stated that the basis of reconciliation was embodied in a series of resolutions which would be introduced by Dr. Logan and himself, as agreed upon by the parties most interested, and he hoped that they would, without discussion, be unanimously adopted by the body as the completion and consummation of the so much desired object.

Whereupon, Dr. J. P. Logan introduced the following, which was adopted, with *one* dissenting voice:

*Resolved,* That the following construction shall be placed upon the action of the Association at Americus, namely—all intention to charge upon the members of the Association personal motives in the then pending controversy is disclaimed, and also any intention to set a precedent for any mutilation of the records of the Association is likewise disclaimed.

*Resolved,* That we do regard the meeting of the Georgia Medical Association of 1868, in Augusta, as a regular meeting of that body.

Dr. Battey then offered the following resolution, which was carried, with *one* dissenting voice:

*Resolved,* That the aforesaid resolution (last above), offered by Dr. Logan, be appended to the official letter of Dr. Boring, of April 10, 1869, and published by the Secretary in the two medical journals and one newspaper at Atlanta.

The following is a copy of the letter of Dr. Boring referred to in the resolution of Dr. Battey—extracted from the printed "Transactions" of the meeting of the Association held in Savannah, April 10, 1869, page seven:

ATLANTA, GEORGIA, April 10, 1869.

At a meeting of the Faculty of the Atlanta Medical College of 1868, held in the city of Atlanta, March 16, 1869, the following resolution was unanimously adopted, viz:

"*Resolved*, That this Faculty disavow any purpose to reflect upon the Medical Association of Georgia, either in the 'Memorial' presented to the Legislature of 1868 or upon any other occasion; and that our representatives, who may attend the meeting of said Association to be held in the city of Savannah, on the 14th instant, be, and the same are hereby, instructed to present this disavowal, together with that contained in the 'Memorial.'

"By order of the Faculty. JESSE BORING, M.D., Dean.

"W. S. ARMSTRONG, M.D., Secretary."

On motion of Dr. Griggs, the meeting adjourned until eight o'clock P.M., with the agreement that the evening session shall be devoted to the discussion of medical subjects.

EVENING SESSION.

The meeting was called to order at eight o'clock P.M.—Dr. McDowell, President, in the chair.

Dr. C. B. Nottingham, of Macon, presented, through Dr. Burgess, the report of a case of *osseous ovarian tumor*, with operation of ovariotomy—successful. Read by Dr. Burgess, and referred to the Committee on Publication.

Dr. Robert Battey, of Rome, Chairman of Committee on Surgery for the Seventh District, submitted a most interesting report, and with it presented and read several reports of cases of interest occurring within his own practice, including one case of successful ovariotomy. Report received, and referred to the Committee on Publication.

On the reports of these cases, an interesting discussion followed, participated in by many members of the profession.

On motion, the Association adjourned until ten o'clock tomorrow morning.

---

COLUMBUS, GEORGIA, April 11, 1872.

The Association met at ten o'clock A.M.—Dr. McDowell, President, in the chair.

Among the portraits placed in the hall, in the decorations for the occasion, is one of General Stonewall Jackson, above the President's chair; to the left, one of General R. E. Lee; to the right, one of General Joseph E. Johnston; and directly under it, one of Dr. A. J. Foard, Medical Director of the Army of Tennessee, who died two years ago.

On motion of Dr. E. J. Kirkscey, a committee of three was appointed to audit the Treasurer's report. Committee—Drs. Rudisill, Philpot, and Walker.

Committee on Nominations was appointed, as follows—one from each county:

Chatham county, Juriah Harris; Fulton county, W. F. Westmoreland; Bibb county, W. F. Holt; Muscogee county, V. H. Taliaferro; Monroe county, A. H. Shi; Talbot county, W. H. Philpot; Upson county, H. Perdue; Crawford county, K. P. Moore; Floyd county, W. D. Hoyt; Troup county, A. W. Griggs; Pulaski county, T. F. Walker; Cobb county, R. B. Anderson; Baldwin county, W. H. Hall; Coweta county, A. B. Calhoun; Jefferson county, E. H. W. Hunter; Twiggs county, W. O'Daniel; Gwinnett county, A. J. Shaffer; Houston county, M. W. Havis; Burke county, E. L. Crump; Sumter county, S. B. Hawkins.

On motion of Dr. J. T. Johnson, the Committee on Nominations was authorized to determine the number of delegates to the American Medical Association.

Committee appointed to audit, reported the Treasurer's statement correct. Shows a balance on hand of $98 25.

Dr. J. P. Logan, of Atlanta, presented the following as the basis of a paper which he had under consideration, but had been unable to complete. This paper proposes, among other objects, to sustain that portion of the recent medical declaration respecting alcohol, signed by many of the leading physicians and surgeons of London, and circulated throughout Europe and the United States—in regard to which there has recently been a most animated discussion in British and American journals, embracing not only the facts asserted, but the use of this substance in the treatment of disease.

The extract referred to, and which is said to have emanated from Dr. Burrows, President of the Royal College of Physicians, London, is as follows:

"As it is believed that the inconsiderate prescription of large quantities of alcoholic liquids by medical men, for their patients, has given rise, in many instances, to the formation of intemperate habits, the undersigned, while unable to abandon the use of alcohol in the treatment of certain classes of disease, are yet of the opinion that no medical practitioner should prescribe it without a sense of grave responsibility. They believe that alcohol, in whatever form, should be prescribed with as much care as any powerful drug, and that the directions for its use should be so framed as not to be interpreted as a sanction for excess, or necessity for the continuance of its use when the occasion is past."

### *Title of, with Propositions Contained in, a Paper by J. P. Logan, M.D., of Atlanta, Georgia.*

Title: "False Therapeutics and Practice as a Cause of Intemperance."

Propositions or divisions contained in paper:

1. The discovery and acknowledgment of error is the first step toward reformation, embracing an assertion and consideration of scientific and popular errors in regard to the use of alcohol as a hygienic and remedial agent.
2. False therapeutics and practice in the use of alcohol as a remedial agent in acute disease.
3. False therapeutics and practice in the use of alcohol as a remedial agent in chronic pulmonary disease.
4. False therapeutics and practice in the use of alcohol as a remedial agent in indigestion.
5. These errors furnish fruitful sources of disease and intemperance.

Referred to Committee on Publication.

Dr. E. J. Kirkscey proposed the names of J. O. Hunt, T. A. Raines, and G. W. Brooks, as permanent members of the Asso ciation. Carried. Other members were also elected, but we failed to get their names.

Dr. Love, of Atlanta, read a paper on "Hemorrhagic Malarial Fever," giving his experience in its treatment, and his views regarding its origin, especially the pathology and mode of treatment. Referred to Committee on Publication.

Dr. Ray, of Atlanta, offered the following, which was referred to a committee of three, to report this afternoon:

WHEREAS, It is desirable that there shall be a general meeting of the medical officers of the Confederate States army and navy, for the collection and preservation of such facts as tend to the promotion of our science; be it

*Resolved*, That this Association suggest that such meeting take place in Atlanta, on the first Wednesday in November, 1872, and that the President and Corresponding Secretary adopt the means most available for communicating this action to the medical men above referred to.

Dr. Stout (late Medical Director of the Confederate States army) and others objected, on the ground that such a convention would be undesirable unless it could be general. He had refused, and would continue to do so, all information to local bodies or individuals, until some organization, general in charter, was called into existence, which would do justice to the medical staff of the late Confederate States army.

Referred to a committee of three—consisting of Drs. E. S. Ray, Robert Battey, and G. Rudisill—to report this afternoon.

On motion of Dr. DeCortez, of Savannah, the action on the paper of Dr. Th. Schumann, of Atlanta, read yesterday, was reconsidered.

After some remarks by Drs. Battey, Stout, and Thomas, on motion of Dr. DeCortez, the paper was received without further action.

The Committee on Nominations reported the following nominations of officers for the ensuing year:

*President*—G. W. Holmes, of Rome.
*First Vice-President*—Juriah Harris, of Savannah.
*Second Vice-President*—A. W. Griggs, West-Point.
*Corresponding Secretary*—W. C. Musgrove, Burke county.
*Treasurer*—W. O'Daniel, Twiggs county.
*Orator*—J. T. Johnson, Atlanta.

Also, the following delegates to the American Medical Association:

Juriah Harris, R. D. Arnold, Savannah; W. F. Westmoreland, J. P. Logan, E. S. Ray, Atlanta; W. H. Doughty, Augusta; E. J. Kirkscey, V. H. Taliaferro, J. E. Bacon, Columbus; S. G. White, Milledgeville; Robert B. Ridley, LaGrange; C. D. Smith, Newnan; W. O'Daniel, Twiggs county; E. F. Walker, Pulaski county; P. H. Wright, Macon; S. B. Hawkins, Americus; A. W. Griggs, J. S. Todd, West-Point; G. M. McDowell, Barnesville; Robert Battey, W. D. Hoyt, Rome; W. A. Greene, Americus; C. B. Nottingham, Macon; H. F. Campbell, Augusta; A. B. Calhoun, Newnan; F. A. Stanford, Columbus.

Dr. C. Terry, of Columbus, presented and read a report of a very interesting case of *superfœtation;* and also a report of three very remarkable cases of gun-shot wounds of the brain, showing to what extent the brain may be injured and recovery follow. The reports were received, and referred to the Committee on Publication.

Dr. Wm. Abram Love, Chairman of Committee on Surgery from the Second Congressional District, submitted a report, which was referred to the Committee on Publication.

The following new members were elected:

S. Richardson, T. M. C. Rice, Macon; Thomas Gibson, W. G. Westmoreland, Hamilton; N. Wells, Talbotton; J. C. Hunt, Barnesville; T. J. Collin, Colaparchee; G. W. Brooks, Lumpkin; T. A. Raines, Jamestown; T. B. Murchison, Hickory Grove.

Dr. K. P. Moore read a paper on bromide of potassium, showing its beneficial effects in cerebral diseases of childhood. Referred to Committee on Publication.

Dr. Thomas, of Savannah, by request, addressed the Association on the subject of bromide of potassium. He warmly advocated its use in many cases—dropsy, Bright's disease, etc.—giving the history of a case in illustration of his views. He took occasion to compliment Dr. Moore, the young gentleman who had read the paper on the subject.

The President called the attention of members to the fifth by-law, and invited them to visit the Secretary, register, and pay up their dues. The response appeared to be very general.

On motion of Dr. Battey, the assessment this year was fixed at two dollars. The Treasurer was afterward authorized to make an additional assessment, if necessary to defray the expenses of the Association.

Dr. E. F. Colzey stated to the Association that all members had not received invitations to the entertainment to be given to-morrow night, because the list of members had not been completed. Members would find their invitations on the Secretary's desk. He hoped all would remain and partake of the tendered hospitality.

On motion of Dr. Battey, the Secretary was instructed to publish minutes as he takes them, and, besides, publish contributions in medical journals.

Amended by Dr. Logan so as to allow authors to select the journal. Adopted.

The Association declined the invitation of the Mayor to visit the public schools of the city, as business at that hour (eleven A.M.) was too pressing, but would accept at an hour of leisure.

The report of Dr. F. A. Stanford, Chairman of Committee on Surgery for the Third District, was read by the Assistant Secretary, Dr. J. T. Johnson. Several cases, with treatment, were reported. Dr. Stanford occupies a proud position among leading surgeons, and his reports were listened to with marked attention.

Drs. Shaffer, of Gwinnett county, and W. F. Westmoreland, of Atlanta, verbally reported surgical operations for imperforate anus. Dr. Battey also mentioned cases he had observed in Paris.

Dr. A. J. Shaffer exhibited some instruments he had invented for the removal of foreign bodies from the uterus and the œsophagus.

Dr. Griggs made suggestions about an expedient he had invented out of bonnet-wire for the removal of placental fragments, etc., from the uterus, which could be extemporized in a very few moments, and serve an admirable purpose in the absence of better instruments. It consisted of a wire twisted and bent upon itself, leaving the upper end in the form of an open hook.

Dr. Jelks mentioned that he had found quinine useful as a parturient.

It was decided that the oration of Dr. Griggs be delivered to-morrow, at eleven A.M.

Dr. Terry, being called upon, reported a case of cancerous breast, removed after extensive ulceration had taken place; interesting from the fact that, although the rule is not to operate in such cases, the advantage derived justified the operation. The woman lived twelve months free from pain, the wound being healed by the first intention, after which the disease returned, and death followed a short period of suffering.

The report of the Chairman of the Committee on Surgery for the Fourth District—whose name we failed to note—was received, read, and referred to the Committee on Printing.

Dr. C. D. Smith reported a case of puerperal convulsions treated by bromide of potassium and chloral hydrate, without success. He then resorted to hypodermic injections, with similar effect. A resort to the lancet proved successful.

Dr. Stout addressed the body in reference to this case, urging that too blind adherence to new chemical and fanciful physiological theories was leading physicians away from old-established landmarks, while the settled experience and positive results were ignored.

Our space and the character of the subject will not permit us to follow, as we would like, the interesting remarks of Dr. Stout. They were listened to with marked interest by those present, and he was frequently applauded.

Dr. Bacon reported a case which confirmed the truth of Dr. Stout's remarks.

Dr. DeCortez stated that he never bled, but used chloroform.

Dr. Griggs gave his experience, and advocated a mean between the theories of Drs. Stout and DeCortez.

Dr. Stout's idea was to bleed enough to *impress* the patient.

Dr. Taliaferro expressed opinions at variance with those of Dr. Stout.

On motion of Dr. Westmoreland, it was resolved, that Dr. Stout be requested to furnish his views for publication.

The members were again requested to enroll their names, that all might be given invitations to the banquet.

At half-past one o'clock P.M., adjourned to meet at three.

### AFTERNOON SESSION.

The Association met at three o'clock P.M.—Vice-President Kirkscey in the chair.

The discussion of puerperal convulsions and blood-letting was resumed by Dr. Stout, who enlarged and explained his views upon the pathology of the disease.

Dr. C. D. Smith supported his view.

The discussion was continued by Dr. Shaffer, and also by Dr. W. A. Greene, the last of whom made mention of veratrum by hypodermic medication, which he regarded as far better than any method known for any kind of convulsions. Veratrum he used frequently in the treatment of pneumonia and pleurisy.

Dr. DeCortez moved that this discussion be postponed for the present, in order to hear the report from the Committee on the Constitution.

Dr. Griggs thought it would be better, on account of the absence of some of the committee, to defer the report until the next meeting of the Association.

The report of the Committee on Constitution was postponed to next session.

Dr. DeCortez offered an amendment to the Constitution, which provides that before a person can become a member of the Association, he must have been a graduate of a college or university in good standing; must be in good standing with the local medical society—or if not, have five indorsements from nearest one—and pass an examination before a committee appointed by the Association. Referred to the Committee on Constitution.

Dr. E. S. Ray, Chairman of the special committee, reported as follows, which was adopted:

*Resolved by the Georgia Medical Association,* That in order to the initiation of an attempt to secure the performance of the important duty of preserving the results of the medical and surgical labors by the medical staff of the Confederate army and navy, this Association respectfully calls the attention of other medical associations in the South to the necessity of a convention of all medical officers, to devise some means of preserving and publishing to the world the result of their professional experience during the late war of the States.

*Resolved,* That this Association suggests Atlanta, Georgia, as the place, and ——— as the time for said Convention.

*Resolved,* That ——— are hereby appointed a Committee of Arrangements, whose duty shall be to correspond with other associations, and with medical officers of our army and navy, and to do anything which may in their opinion tend to secure a large attendance at the proposed convention, and to promote the laudable object of its assembling.

Dr. E. J. Kirkscey offered the following resolution:

*Resolved,* That the druggists, pharmaceutists and chemists of the State be requested to call a convention at an early day, and organize a State Pharmaceutical Association, to meet annually, at the same time and place of the Medical Association, and co-operate with it in all measures of mutual interest and importance.

Dr. E. J. Kirkscey introduced a resolution that the Association recommend and urge the State Government to adopt and enforce a systematic registration of marriages, births and deaths, and that a standing committee of three be appointed by this Association to take general charge of the subject, and report to this Association. Adopted.

A motion by Dr. Kirkscey, to memorialize the Legislature to establish a Vaccine Bureau, with a salaried agent, was laid on the table.

Committees on Practice of Medicine and Surgery for several Congressional Districts were heard, and reports received.

Several Committees on the Practice of Medicine and Surgery were discharged.

Dr. Love offered the following, which was adopted:

*Resolved*, That the President of this Association appoint from each Congressional District of the State a committee of three on the Practice of Medicine, and a committee of three on Surgery, who shall report on some special subject or subjects, or make report of cases at their discretion, and present the same at the next meeting of the Association, and that individual members be requested to make individual reports in like manner.

Dr. W. F. Westmoreland presented a report on the hæmostatic properties of the muriated tincture of iron in the arrest of secondary hemorrhage, and as a substitute for the ligature in the case of hemorrhage from the larger arteries, with the report of a case, and experiments on the lower animals; also its successful application in a case of aneurism of the axillary artery. Paper referred to Committee on Publication.

Dr. Griggs, of West-Point, made some remarks upon the same subject, and mentioned the good results following the application of matico to bleeding gums. Dr. Bacon also added his testimony.

It was announced that standing committees were discontinued on account of expirations of term of office.

Dr. Westmoreland made some remarks concerning his experience in the return of malignant diseases.

Dr. Orme asked the Association to hold its next meeting in Atlanta; Dr. Cortez, in Savannah. Ruled out of order at the time.

Dr. Smith made some remarks concerning epidemic meningitis.

Meningitis was discussed by Drs. Griggs, Stout, J. G. Westmoreland, and Hoyt.

Dr. L. A. Dugas, of Augusta, a former President of the Association, and Professor in the Augusta Medical College, was introduced and invited to the stand occupied by presiding officers, amid much applause. He accepted the invitation.

Savannah, Atlanta and Rome were put in nomination for the next place of meeting. Atlanta was selected by a large majority.

The Association adjourned to eight o'clock P.M.

NIGHT SESSION.

The Association met at eight o'clock P.M.—the President, Dr. McDowell, in the chair.

The following standing committees were appointed from the various Congressional Districts:

FIRST DISTRICT.

*Practice of Medicine*—R. D. Arnold, J. J. Dearing, Chatham county; J. M. Cargil, Glynn county.

*Surgery*—T. J. Charlton, C. Ganahl, R. J. Nunn, Chatham county.

*Gynæcology*—J. S. Minnus, H. H. Smith, Scriven connty; J. B. Reid, Chatham county.

SECOND DISTRICT.

*Practice of Medicine*—J. E. McMillan, Dougherty county; W. A. Strother, Mitchell county; P. L. Hilsman, Dougherty county.

*Surgery*—B. M. Cromwell, W. L. Davis, Taliaferro Jones, Dougherty county.

*Gynæcology*—G. F. Cooper, W. A. Greene, William Hardwick, Sumpter county.

THIRD DISTRICT.

*Practice of Medicine*—J. E. Bacon, Columbus; K. P. Moore, Crawford county; R. B. Ridley, LaGrange.

*Surgery*—William H. Philpot, Talbot county; T. T. Smith, M. W. Havis, Houston county.

*Gynæcology*—V. H. Taliaferro, F. A. Stanford, Columbus; T. C. Ellison, Talbot county.

FOURTH DISTRICT.

*Practice of Medicine*—J. J. Caldwell, Pike county; A. H. Shi, Monroe county; C. H. Hall, Bibb county.

*Surgery*—D. W. Hammond, Bibb county; B. F. Rudisill, Monroe county; C. S. Strother, Pike county.

*Gynæcology*—J. T. Banks, Spalding county; W. F. Holt, J. R. Boon, Bibb county.

FIFTH DISTRICT.

*Practice of Medicine*—H. H. Steiner, Richmond county; E. H. W. Hunter, Jefferson county; M. P. Deadwyler, Elbert county.

*Surgery*—L. A. Dugas, Richmond county; A. G. Whitehead, Burke county; T. L. Satterstedt, Richmond county.

*Gynæcology*—W. H. Doughty, S. C. Eve, Richmond county; H. L. Battle, Jefferson county.

SIXTH DISTRICT.

*Practice of Medicine*—R. D. Moore, Clarke connty; B. F. Carleton, Greene county; M. V. Aderhold, Oglethorpe county.

*Surgery*—H. H. Carlton, M. F. Howard, Clarke county; A. J. Shaffer, Gwinnett county.

*Gynæcology*—R. M. Smith, C. W. Long, Clarke county; T. H. Hutchinson, Oglethorpe county.

SEVENTH DISTRICT.

*Practice of Medicine*—E. S. Ray, J. M. Johnson, Fulton county; W. D. Hoyt, Floyd county.

*Surgery*—W. F. Westmoreland, Atlanta; Robert Battey, Rome; A. B. Calhoun, Newnan.

*Gynæcology*—J. P. Logan, Atlanta; D. G. Hunt, Gordon county; J. T. Jelks, Cobb county.

A motion of Dr. Kirkscey, that the roll of membership be called for the purpose of correction, was adopted.

On motion of the President (the Vice-President in the chair), the rules were suspended to hear Dr. G. N. Holmes on the death of Dr. A. J. Foard, Medical Director of the Army of Tennessee.

The Doctor pronounced the deceased a noble, high-toned, honorable gentleman, and a good surgeon. He alluded to his close connection with the heroes whose likenesses were grouped on the wall—Generals Lee, Johnston, and Jackson—in the Mexican and late war. The Association should pay him the tribute of respect.

Dr. Stout pronounced a brief eulogy on the dead. He illustrated the arduous duties of the surgeon in the field in the Confederate army, and showed that they deserved as much credit as any officer. No country had ever a finer medical staff than the Confederate States. Dr. Foard was peculiarly well-qualified to organize such material. He was able to choose his men, and to systematize officers taken from civil life. These faculties made him great. To make himself and those under him useful in the highest degree, was his purpose. Some one will yet write a history who will do justice to the medical department of the Confederate States.

Dr. Logan said the deceased illustrated one of the highest elements of human nature—self-sacrifice. He had told him (Dr. Logan) that when he resigned his brilliant prospects in the

United States army, he felt satisfied the Confederacy could not succeed. His death was the result of the same spirit, in the determination to discharge the duties of a professorship to which he had been elected in Baltimore, while his health was not adequate to the task.

President McDowell was highly eulologistic of his administrative qualities. Especially was this manifested in the Hood compaign, and it was exhibited in such a manner as to win the love of all subordinate officers.

Dr. Logan moved that a committee of five be appointed to adopt suitable resolutions regarding Dr. Foard, and that Dr. G. N. Holmes be the chairman. Adopted.

Drs. Holmes, Stanford, Stout, Logan, and W. F. Westmoreland, were appointed that committee.

In honor of the dead, Dr. Kirkscey moved an adjournment to nine o'clock to-morrow morning. Carried.

---

Columbus, Georgia, April 12, 1872.

The Association met at nine o'clock A.M.—Vice-President Kirkscey in the chair.

The session was opened with prayer by Rev. Dr. Arminius Wright, of St. Paul's Church.

Calling of the roll was resumed, when the following names were entered on the roll of the dead:

S. A. Billing, S. H. Dean, M. A. Franklin, William Frazer, W. L. Felder, J. B. Ficklin, A. Gaither, E. Girardey, G. M. Gordon, H. K. Greene, A. B. Greene, J. B. Gregory, A. B. Gregory, T. O. Hugg, G. M. Harrison, E. Hoopes, J. C. Habersham, Stephen Habersham, W. L. Jones, J. O. Jones, R. H. Lockhart, J. G. McGee, J. R. Alexander, J. J. Boswell, H. L. Battle, J. W. Beeson, H. A. Bignon, R. C. Black, D. J. Bothwell, William Burns, B. F. Bomar, F. S. Colly, J. C. Carroll, W. D. Cunningham, R. Q. Dickinson, L. J. Dupree, J. F. Duckinson, Thomas Hoxey, T. O. Heard, E. B. Hook, W. S. Lightfoot, R. McGouldrich, C. A. McKinley, R. A. Nash, H. J. Ogilby, D. B. O'Sullivan, J. D. Owen, J. M.

Parsons, G. W. Pitts, T. L. Rives, R. A. T. Ridley, James Rugan, —— Rusk, J. N. Simmons, J. D. Smith, H. Steele, W. B. Stephens, J. W. Shropshire, W. A. Spier, E. T. Taylor, Charles Thompson, A. A. Trammel, Gilbert Tennant, Charles West, J. B. Wiley, G. A. Winn, C. T. Woodson, W. H. Wooten, J. A. Wragg, E. O. Ware.

Thus far forty-nine members are reported dead since the first organization of the Association.

Dr. Kirkscey offered the following, which was adopted:

*Resolved,* That any member of the Georgia Medical Association, who has been, or shall be, expelled by any local society in connection with the Georgia Medical Association, for any violation of the Code of Medical Ethics, shall be suspended from membership in this Association until an appeal is taken, or a reversal of decision of said society.

Dr. J. Stainback Wilson, of Atlanta, read a synopsis of an interesting and novel paper on parturition not necessarily a painful process, with some suggestions as to the hygienic treatment of pregnant women as a means of mitigating the pains of labor. Referred to Committee on Publication.

The committee appointed to draft a testimonial to the late lamented Andrew J. Foard, M. D., Medical Director of the Army of Tennessee of the Confederate States, made the following report:

WHEREAS, No formal notice by any medical organization has been taken of the death of Andrew J. Foard, M.D., Surgeon and Medical Director of the late Confederate army, and this body being forcibly reminded of the failure to do justice to the memory of the honored and lamented dead by this visit to his former home;

1. *Resolved,* That the Georgia Medical Association deeply lament the early death of A. J. Foard, who illustrated in his life some of the noblest traits of humanity, viz—self-sacrifice and exalted patriotism in the surrender of his enviable position and prospects as a medical officer in the United States army under a sense of duty to his native State in the time of peril, which shall stand as a monument to his memory.

2. *Resolved,* That as a medical officer, in the most trying and responsible positions during the late war, he was ever equal to the emergency, and discharged his duties with signal ability.

3. *Resolved,* That we will ever hold the memory of A. J. Foard in our most sacred remembrance for his noble traits as a man, his ability and faithful discharge of duty as a member of our profession, his eminent success as a medical officer under the most trying circumstances, and his gallantry as a soldier.

4. *Resolved,* That a copy of this preamble and resolutions shall be transmitted to his surviving relatives by the Secretary of this body.

5. *Resolved,* That the Secretary be directed to devote a page or more, as may be deemed necessary, of the transactions of this body, to the memory of the deceased.

Dr. Terry said that his long association with Dr. Foard rendered it proper that he should offer a few remarks. His connection extended over the entire term of Dr. Foard's connection with the Army of Tennessee, from Pensacola, through the Mississippi, Kentucky, and Tennessee campaigns. Dr. Foard's power of organization was very great. Educated, like all army officers, to look upon the Regulations as the perfection of wisdom on the subjects of which it treats, yet he said, "It was not written for such a war or such an army," and red tape never prevented him from taking any responsibility that would benefit the army. His devotion to the cause was equaled only by his sacrifices. Dr. Terry heard Dr. Foard say at an by all the old army officers, who predicted exactly the mode early date in the war, when all looked bright, and the hope, and even certainty, of success warmed the heart of every Confederate, that we *must certainly* fail; and in this he was joined and causes of failure, of which they were equally convinced at the moment they tendered their resignations, and from a sense of duty gave up certain promotion and a life-long office to join a cause they *knew* must fail. To his knowledge of men, as shown in the selection of officers, is due the high reputation of the medical staff under him. His gallantry was conspicuous. Having placed his hospitals in the hands of those he knew would properly conduct them, he always accompanied his General to the field, and while watching over him, discharged all the duties of a staff officer; and every one knows that he who kept with Joseph E. Johnston and Braxton Bragg, on the battlefield, had no safe place. General Johnston had an unhappy faculty of getting in the way of the enemy's balls, and at Shiloh, General Bragg had two horses shot under him; Dr. Foard was among the first to assist him to remount.

Dr. Kirkscey said that Dr. Foard was the private soldier's friend.

The resolutions were adopted by a rising vote.

Dr. George N. Holmes reported that twenty-eight years ago, in Kentucky, he delivered a poor white woman of six babes at one birth. The children were perfectly formed, and weighed each two and a half to three and a half pounds. The parents were originally from Maryland. They afterward moved to Arkansas, and he had heard that two of the children had died.

Dr. Love offered the following, which was adopted:

*Resolved,* That the thanks of the Association are due and are hereby tendered to the officers of the Western & Atlantic Railroad, Rome Railroad, Macon & Western Railroad, Selma, Rome & Dalton Railroad, Macon & Brunswick Railroad, Georgia Railroad, Atlanta & Richmond Air-Line Railroad, Atlanta & West-Point Railroad, Western Railroad, and Southwestern Railroad, for their liberality in passing members to and from this meeting at half-fare; to the press of this State generally, and Columbus especially, for their courtesies; to the proprietor of the Rankin House for the use of his Skating-Rink Hall and the supply of lights free of charge; to the Medical Society of Columbus, the municipal authorities and citizens of Columbus, for the hospitable manner in which this Association has been received and entertained during its stay in the city.

*Resolved,* That the Secretary be requested to give publicity to the above resolution through the press.

Dr. Love read a tribute of respect to the late Dr. J. N. Simmons, of Atlanta.

Dr. G. W. Holmes moved that Dr. Love be added to the Committee on Necrology. Carried.

Dr. Logan moved that the names of all the dead be referred to that committee, and that their report be referred to the Committee on Printing. Adopted.

Dr. Colzey offered a resolution appointing certain hours to be permanently appropriated, at each annual session, to the subject of necrology, as follows:

WHEREAS, It is eminently proper that the demise of members should receive proper notice at each meeting of the Association; therefore, be it

*Resolved,* That the hours of from twelve to half-past one o'clock, the second day of each session, be appropriated to the reading of memorial papers—which papers shall be reported to the Committee on Necrology.

Referred to a Special Committee on the Constitution.

Dr. Taliaferro offered some specimens of uterine cloth tents, of his invention, and a paper descriptive of the same, which was listened to with great interest.

Dr. Logan desired to add his testimony to the great value of the cloth tents. He also called attention to the value of the extract of pinus canadensis, used upon the tent in endometritis, referring to the fact that Dr. J. Marion Sims, of New York, had introduced the article to the profession as an application to the vagina and os uteri.

Dr. W. F. Westmoreland followed Dr. Logan, with a full indorsement of Dr. Taliaferro's tents.

Dr. George M. McDowell offered the following, which was adopted:

*Resolved,* That the thanks of the Association are due, and are hereby tendered, to the Mayor of the City and the Superintendent of the Public Schools of Columbus, for the invitation to visit the schools, with regret that the unusually pressing duties of the body prevented an acceptance of the same.

Dr. Word reported a case of a young lady, of Columbus, in whom quinine always produces a disease like scarlet fever.

Dr. Love gave two cases in which quinine produced similar effects.

Dr. Starbuck related a case of Dr. Names', in which quinine produces somewhat similar effects, and that Dr. Names judges of the probable duration of the case by the length of time it requires to produce these effects.

Dr. E. F. DeGraffenried called attention to the death of his father.

Dr. Logan offered the following, which was adopted:

*Resolved,* That the Committee on Necrology report upon the names of members who have died, which have been brought to the attention of the Association, and that the special papers in regard to such individuals be referred to the same committee, and that they report at this meeting, to be referred to the Committee on Printing, with power to print.

The resignation of Dr. S. H. Stout, as Permanent Secretary of the Association, was read. He resigned because of a press of business.

On motion of Dr. Logan, the resignation was, unanimously, not accepted. Highly complimentary to Dr. Stout.

Dr. Greene moved a short recess, preparatory to the address of Dr. Griggs. Carried.

After a recess of ten minutes, the Association was called to order by Dr. McDowell.

The hour having arrived for the delivery of the annual oration, Dr. Griggs was introduced, and spoke as follows:

Mr. President,—The happiness of mankind has been enhanced by the brilliant discoveries of the physicist, who has unfolded some of the transcendent beauties of nature, and explained the character and distinctive features of the laws which control materiality. The diffusion of knowledge—elucidating intricate and difficult problems everywhere abounding—has dignified the intellect, refined the taste, and exalted the affective faculties of the soul. The fogs of superstition are clearing away as the sunlight of science is glittering near by; and its radiant beams are ready to rest, even now, on the sky that spans the land of the deluded idolater. Chance nor accident belong to God. He pronounced His work good when it was finished, and placed it under the control of statutory provisions ordained for its government. It is known that there are certain definite laws which operate in the physical world, producing various phenomena, often observed, less often eliciting attention as to the relations of cause and effect, though they act with unvarying constancy and determinate results. Matter constitutionally possesses an inherent power or attractive force, existing between the atoms of individual substances, developing forms according to kind—the aggregate force of whose particles, proportioned to the quantity of matter, establishes the relations present between every planet, superincumbent and surrounding object, and between the planets themselves. The same force displays itself in the germination, growth and perfection of plants, and in the nutrition, development and maturation of man and animals. Molecular or chemical, magnetic or electrical, are but different expressions of the same power.

The greatest minds have not yet fathomed the depths of this subject beyond the fact that attraction is an inherent property of matter, organic and inorganic. A missile is hurled in the air; it is retarded regularly in its ascent, finally checked, and descends, with constantly increasing velocity, to earth. Why did it not continue forever in the direction in which it was projected? Because of the superior force of the earth's attraction. That is all; no one can tell more. We may say *because*, *because*, like a child questioned above its comprehension, but the actual great reason defies research. We may understand a law in its simple or multiplied effects—direct, indirect, or remote—and still may not be able to comprehend the physical wherefore of its authority. We refer to the well-known law of gravitation; its instahtaneous, its uniform action, proportioned to the quantity, but independent of the quality of matter, is not affected by the intervention of other bodies, however solid or liquid, dense or aeriform—acting in all directions from the center of the globe to incalculable distances beyond the surface—decreasing in force as the square of the distance increases, and holding the planets within their proper spheres as they roll in endless grandeur through the realms of illimitable space. The light of science illuminates the subject, and experimentally verifies the facts announced, and still we can not discover why this property universally attaches to matter. The rainbow's diamond hues, grandly arched in the heavens, find their counterpart in the rays of the solar spectrum. That light is decomposed in its passage through the rain-drop, is proven by the prism, but who knows why? Different media, and the greater or less obliquity at which the rays enter them, affects definitely the phenomena of refraction, and different rays possess different degrees of refrangibility, though we are unable to solve the mystery, unless we ascribe it to the belief that light is material, and therefore influenced by gravitation. The aurora, with imposing beauty, canopies with golden clouds the regions of the North, and sometimes blushes in the far South. They tell us that this beauteous queen is only electricity waving martial banners in pure thin air. What is electricity? A subtle agent, imponderable, of the greatest known velocity—attracts and repels—is sometimes positive, then negative—is only a condition of matter, created by the Father for His own wise purposes.

We need not multiply examples to prove our inability to solve the great problem of nature. The untiring mind of the philosopher has traversed the subject from atom to atom, individual to individual, and from world to world, and at last, confused in thought and overpowered in understanding, been constrained to acknowledge, with humility and reverence, that God has hidden from human knowledge the divine essence of natural laws.

Physics is the grand tributary to the Science of Medicine. Their waters mutually blend and commingle harmoniously together as they bear upon their bosom the accumulated store of the costliest jewels found in the great depths of the past. Man is not exempt from the action or effect of physical law; he is as subject as a microscopic molecule or a revolving planet. Created from the dust, he inherits its power, and exhibits in his economy the analogue of all the elements of inorganic nature. Molecular change marks every respiratory act, every contraction of the heart, and every mental exertion and emotion, and personal identity is only preserved by constancy in the arrangement of the atomic particles constituting the physical being.

Rocks disintegrate, metals fuse, and chemical compounds are dissolved by the power of heat. Heat superinduces morbid changes in the human system, developing inflamations involving molecular tissues in ruin, sometimes eventuating in somatic death. The life of plants is preserved by an elective affinity exercised by rootlets upon decomposed matter, and by respiration through their leaves. The human body is sustained through the agency of the same principles. Light is essential to the health and vigor of vegetation; so it is to that of mankind. The galling chain of the criminal does not dispirit half so much as doth the darkness of the prison cell. The forces which belong to external nature also belong to man's being, and, with the addition of nerve-force, bear the same correlative and conservative relations to each other. Chemical compounds dissolve under the play of new affinities, metals oxidize and fall to powder, and as for the man, the irrevocable decree is published—"Dust thou art, and unto dust shalt thou return."

It is needless to further pursue the parallel. Ere man violated the holy trust, and the blighting curse of Jehovah fell upon him, he was perfectly adapted to the situation, so that he maintained an equilibrium under the active forces in and around him, whilst he breathed the pure atmosphere of Eden, fed upon her delicious fruits, drunk her crystal waters, and oft regaled himself with the perfume of sweetest flowers, as he reveled amidst fruitful vines and sylvan bowers. Spiritual death came, and appalling as it was, through the Providence of a Saviour, the portal was opened for a new life infinitely above our comprehension. Physical death began, equilibrium was lost, and the hygiene of Moses was insufficient to arrest the calamities of disease. The mind was perverse and dark in those ancient times; but self-preservation and protection exercised the ingenuity in attempts at art, until many difficulties were overcome, and conveniences and comforts secured. Philosophers began to reason upon the problem of the universe, but their theories were built upon mythical hypotheses, for they tried to "reason out all things from the depths of the soul." Ages past before the observation of facts resulted in the system of experimental philosophy which disclosed the principles of inductive reasoning. This was the inception of the grand scheme which broke open the vaults of Nature, and declared the uniform impress of Divinity upon all things. Long-imprisoned truths were liberated, and sent on swift charities to the learned of every land. The Science of Medicine was born, partially asphyxiated by the lingering smoke of superstition, and was wrapped in a swaddle of fancy and diverse colors; it grew slowly for the want of proper attention and nourishment, and its form bent forward under the weighty dogmas of pioneer philosophy. It was the faint hope of the race then, and it is the greatest earthly boon to-day. The identity of the elementary principles of the inorganic and vegetable with those of the animal kingdom, in which man holds the highest rank, is at once suggestive of the important fact that they are reciprocally transferable, under circumstances favoring the unembarrassed play of the attractive forces common to both. Whilst we believe this to be the fundamental idea of medicine, we do not ignore the fact that many things in connection with the subject are yet full of mystery. We have not perhaps discovered all laws exercising important functions in the world around, above, and beneath us. This we do know, that all the physical forces yet discovered operate on or reside in the human body, and are essential to its preservation; that the elements of inorganic matter are disposed differently to the tissues producing variety in structure, and therefore the essential diversity of functions by the different organs. The Science of Medicine is not the mere compilation of experimental results; it grows out of the very nature of things; its foundation is in a knowledge of physics, including anatomy, in which the uniform hand-writing of the Creator shows unity of design in the unequalled architecture of the universe. Then it is a natural science, and the laws which control its wide domain are definite—in a sense, eternal. They are divine in point of athority, and physical in their application and action. When we shall have thoroughly learned their nature, we shall then be able to understand the mutual relations and dependencies subsisting between the physical and the psychical, and the practice will conform to the principles involved, and Medicine will bear her colors aloft, inscribed in characters of living light—*Lex Naturæ Lex Dei.*

On motion, the eloquent address of Dr. Griggs was referred to Committee on Publication.

On motion of Dr. Stout, the welcoming addresses of the Mayor and Dr. Taliaferro were requested for publication.

The following new members were elected:

T. S. Mitchell, Hamilton; J. W. Cameron, Harris county; C. C. Patillo, J. E. McMillan, West-Point; J. B. Baird, Atlanta; W. B. S. Holmes, Rome; A. Matthews, Sandersville; B. F. Chambless, R. F. Wright, Forsyth; E. L. Bardwell, Talbotton.

Dr. Love, from the Committee on Necrology, reported upon the deaths of Drs. Billing, Ridley, and Simmons, and included in the roll in memoriam all those heretofore mentioned. Received and referred.

Adjourned at one o'clock P.M. to meet at three P.M.

AFTERNOON SESSION.

The Association met at three P.M.—President George M. McDowell in the chair.

Dr. Love offered the following, which was adopted:

*Resolved,* That Dr. J. T. Johnson be and is hereby appointed Assistant Secretary, and in the recess of the Association, or in the absence of the Permamanent Secretary, that he be authorized to act in his stead.

Dr. W. F. Westmoreland offered the following, which was adopted:

*Resolved,* That the Committee on Revision of the Constitution be requested to consider the propriety of prohibiting the election of any person to membership who is not present and ready to take his seat in the Association.

Dr. Love, Delegate to the Alabama State Medical Association, lately held in Huntsville, was called on by the President, when he submitted a verbal report, stating that he had been most cordially received and hospitably entertained, and that the Alabama State Association had appointed a delegate to this, but unavoidable circumstances had prevented his attendance. Dr. Love spoke of the Alabama Association in a very complimentary manner, as a well-organized working body of men, of whom the State should feel proud. He reported upon the nature of their organization, the interesting character of their proceedings, and the value of their volume of "Transactions"

to science-loving men, and as material stored for the future medical history of the State.

On motion of Dr. W. F. Westmoreland, it was resolved that a delegate from this Association be appointed by the President elect to the next session of the Alabama Medical Association, which meets at Tuscaloosa, on the last Tuesday in March, 1873.

The Treasurer reported $210 25 in the Treasury.

Dr. A. W. Griggs offered the following:

*Resolved*, That any member who fails to pay his assessments for three consecutive years have his name dropped from the rolls, unless excused by the Association. Referred to Committee on Constitution.

A motion of Dr. Logan, that all members of this Association be notified by the Treasurer of the assessment due from each, was adopted.

A motion of Dr. Terry—that the Secretary be requested to ascertain the cost of publishing full minutes, and if collections by the Treasurer are not sufficient, he make another assessment upon members—was adopted.

Dr. Ray moved that the resolution of instruction to the Committee on Publication shall be so construed as not to prohibit the publication of the papers presented to this Association on medical subjects as part of our transactions. Adopted.

Dr. Logan moved that the privilege of publishing any article presented to this body, through any of the medical journals of the country, is extended to the authors, provided the Committee on Publication pronounce it worthy, with the understanding that the Association be credited with such paper. Adopted.

Dr. R. E. Bacon, of Columbus, offered the following, which was adopted:

*Resolved*, That the thanks of the Association be, and are hereby, tendered to the President, Vice-President, Secretary, and Treasurer, for the faithful and eminently satisfactory discharge of their several official duties.

The Nominating Committee reported the following Standing Committees:

*On Publication*—W. C. Musgrove, W. O'Daniel, S. H. Stout, and J. T. Johnson.

*On Inebriate Asylum*—W. D. Hoyt, E. J. Kirkscey, G. F. Cooper.

*On Prize Essays*—J. G. Westmoreland, J. P. Logan, W. H. Cumming, G. E. Sussdorff, W. R. Burgess.

*On Education*—J. G. Thomas, A. W, Griggs, F. A. Stanford, Robert Battey, J. F. Alexander.

*On Medical Literature*—H. F. Campbell, W. F. Westmoreland, W. H. Goodwin, R. D. Arnold.

*On Climatology and Epidemic Diseases*—W. A. Love, Carlisle Terry, William Duncan, A. L. C. Magruder, R. B. Ridley, W. A. Greene, E. W. Alfriend, A. B. Calhoun, R. D. Moore, J. M. Howell, E. S. Ford, Robert Battey.

*On Necrology*—G. N. Holmes, R. P. Myers, G. F. Cooper, J. J. Mason, W. H. Hall, H. H. Carlton, J. R. McAfee.

*On Arrangements and Reception*—E. S. Ray, J. F. Alexander, W. G. Owen, A. L. Connally, J. M. Boring, C. Simpson, W. N. Judson.

The report was adopted.

On motion of Dr. Love, the President was directed to appoint, at his leisure, a committee of three, as advisory to the Publication Committee.

Dr. Hoyt offered the following:

*Resolved,* That the Secretary, with the addition of a committee of three members of the Association in good and regular standing, be requested to correspond with all members of the Association who may be reported to them, and who are notoriously engaged in pursuing a course in open violation of the Code of Ethics of the American Medical Association, and inform them of the charges made against them, thus affording them an opportunity of clearing themselves, and that the charges, with their replies, be submitted to a future meeting of the Association for their deliberation, if deemed of sufficient importance.

*Resolved further,* That all members of the Association be requested to inform the Secretary in writing of any acts of the kind on the part of its members within their knowledge.

These resolutions were strongly supported by President McDowell and Dr. E. F. Colzey. Dr. Colzey wanted it rigidly carried out. Dr. Love also spoke in its favor.

The resolutions were adopted. Committee—Drs. Hoyt, or Rome; Goldsmith, of Atlanta; Duncan, of Savannah.

The minutes were read for correction and confirmed.

Dr. Bacon, of Columbus, moved that the Association adjourn to meet on the second Wednesday in April next, in Atlanta.

The President and Secretary made a few appropriate remarks, the resolution was put and carried, when the Association was pronounced adjourned, and thus closed the twenty-third session.

[NOTE.—We can not close this report without returning our thanks to the reporter for the Columbus *Sun*, Mr. DeVotie, and to Dr. E. J. Kirkscey—as well as to Dr. S. H. Stout, the Permanent Secretary, and Dr. J. T. Johnson, Assistant Secretary—for their uniform kindness and aid in furnishing the material necessary in compiling the same.

Much of the report has been taken *verbatim* from the Columbus papers—the *Sun* and *Enquirer*—and we deem it but justice to make the announcement here, returning our thanks for the many courtesies extended.—W. A. L.]

